# Protective Effect of Sevoflurane Postconditioning against Cardiac Ischemia/Reperfusion Injury via Ameliorating Mitochondrial Impairment, Oxidative Stress and Rescuing Autophagic Clearance

**DOI:** 10.1371/journal.pone.0134666

**Published:** 2015-08-11

**Authors:** Peng Yu, Jing Zhang, Shuchun Yu, Zhenzhong Luo, Fuzhou Hua, Linhui Yuan, Zhidong Zhou, Qin Liu, Xiaohong Du, Sisi Chen, Lieliang Zhang, Guohai Xu

**Affiliations:** 1 Department of Anesthesiology, the Second Affiliated Hospital of Nanchang University, Nanchang 330000, China; 2 Department of Cardiology, the Second Affiliated Hospital of Nanchang University, Nanchang 330000, China; University of Alabama at Birmingham, UNITED STATES

## Abstract

**Background and Purpose:**

Myocardial infarction leads to heart failure. Autophagy is excessively activated in myocardial ischemia/reperfusion (I/R) in rats. The aim of this study is to investigate whether the protection of sevoflurane postconditioning (SPC) in myocardial I/R is through restored impaired autophagic flux.

**Methods:**

Except for the sham control (SHAM) group, each rat underwent 30 min occlusion of the left anterior descending coronary (LAD) followed by 2 h reperfusion. Cardiac infarction was determined by 2,3,5-triphenyltetrazolium chloride triazole (TTC) staining. Cardiac function was examined by hemodynamics and echocardiography. The activation of autophagy was evaluated by autophagosome accumulation, LC3 conversion and p62 degradation. Potential molecular mechanisms were investigated by immunoblotting, real-time PCR and immunofluorescence staining.

**Results:**

SPC improved the hemodynamic parameters, cardiac dysfunction, histopathological and ultrastructural damages, and decreased myocardial infarction size after myocardial I/R injury (*P* < 0.05 vs. I/R group). Compared with the cases in I/R group, myocardial ATP and NAD^+^ content, mitochondrial function related genes and proteins, and the expressions of SOD2 and HO-1 were increased, while the expressions of ROS and Vimentin were decreased in the SPC group (*P* < 0.05 vs. I/R group). SPC significantly activated Akt/mTOR signaling, and inhibited the formation of Vps34/Beclin1 complex via increasing expression of Bcl2 protein (*P* < 0.05 vs. I/R group). SPC suppressed elevated expressions of LC3 II/I ratio, Beclin1, Atg5 and Atg7 in I/R rat, which indicated that SPC inhibited over-activation of autophagy, and promoted autophagosome clearance. Meanwhile, SPC significantly suppressed the decline of Opa1 and increases of Drp1 and Parkin induced by I/R injury (*P* < 0.05 vs. I/R group). Moreover, SPC maintained the contents of ATP by reducing impaired mitochondria.

**Conclusion:**

SPC protects rat hearts against I/R injury via ameliorating mitochondrial impairment, oxidative stress and rescuing autophagic clearance.

## Introduction

Cardiomyocyte ischemia during acute myocardial infarction, heart transplantation and shock causes anoxia, energy depletion and acidosis. Coronary reperfusion, rapid recovery of blood flow and oxygen supply is a standard treatment strategy to reduce cardiac injury. However, paradoxically, cardiomyocyte injury is prominent upon reperfusion, known as ischemia-reperfusion (I/R) injury [1]. Mechanisms underlying I/R injury are complex, including Ca^2+^ overload, mitochondrial dysfunction, oxidative stress, endoplasmic reticulum stress, activation of autophagy and apoptosis [2–5]. Hence, identification of a specific and effective drug is imperative to protect the myocardium from I/R injury.

Many studies have been carried out with attempt to decrease myocardial I/R injury. Many volatile anesthetics such as sevoflurane, desflurane and isoflurane have cardioprotective effects [5,6]. Since 1975, sevoflurane, highly fluorinated methyl-isopropyl ether, has been used in many surgeries because of its great benefit for induction and recovery [7]. Similar to the cardioprotection of ischemic preconditioning and postconditioning, sevoflurane preconditioning (SPreC) and sevoflurane postconditioning (SPC) have been shown to provide protection against I/R injury [5,8]. SPC treatment does not require any previous information of the ischemic event, which is more convenient than SPreC in the clinical environment. The cardioprotective effect of SPC is probably functioned by the activation of phosphoinositide 3-kinases (PI3K)/protein kinase b (Akt) pathway, reductions in reactive oxygen species (ROS), upregulation of extracellular signal-regulated kinase (ERK) and heme oxygenase-1 (HO-1) expressions and modulation of the activity of pro- and anti-apoptotic pathways [8–11]. However, potential molecular mechanisms for SPC are not fully elucidated, yet.

Autophagy is a catabolic process, through which long-lived cytoplasmic proteins and damaged organelles are degraded by lysosome-dependent mechanisms. Autophagy has been shown to be triggered by energy depletion, oxidative stresses, protein aggregates and damaged organelles [12,13]. The increased expression in class III PI3K and decreased expression in class I PI3K are important to induce autophagy. Vps34 is the single class III PI3K enzyme in mammals [14,15]. Despite many studies have been conducted, it remains controversial whether the activation of autophagy during myocardial I/R injury is protective or detrimental. Some studies have demonstrated that increased autophagy activity is beneficial; however, excessive activation of autophagy induced autophagic cell death in the myocardial I/R injury [5,16]. Excessive autophagic activity results in the elimination of essential organelles and molecules, and contributes to left ventricle (LV) dysfunction and adverse events. Till now, the exact role of autophagy in SPC-induced myocardial protection remains unknown.

Increased evidence supports organelle-specific, selective autophagy of mitochondria (mitophagy) is important for mitochondrial quality [17,18]. Mitochondrial dynamics have been shown to play an important role in cellular function and mitophagy [19]. Maintaining normal mitochondrial integrity and efficiency is an effective strategy to protect the heart against I/R injury. Therefore, the significance of mitophagy in I/R injury has received great attention in recent years. Mitophagy is an endogenous quality control system of mitochondrion; a crucial question has been raised: what relationship between SPC and the I/R-induced mitophagy?

First, we confirmed that SPC is cardioprotective in a myocardial I/R injury in an animal model of this study. Second, we proved the hypothesis that SPC inhibited excessively mitophagic activity and increased the survival of I/R cardiac myocytes. At last, the potential mechanisms for SPC to protect against myocardial I/R injury were investigated.

## Materials and Methods

### Antibodies and reagents

For more detailed information please refer to the Supporting Information (S1 File).

### Animal

All the experiments in this study were approved by the Institutional Animal Care and Use Committee of Nanchang University (Nanchang, China) and in compliance with the Guide for the Care and Use of Laboratory Animals published by the US National Institutes of Health (NIH Publication No. 85–23, revised in 1996). Adult, male Sprague-Dawley rats, weighing 200–230g and of healthy grade were approved by the Committee of the Medical College of Nanchang University. All rats were obtained from the Animal Center of Nanchang University.

### Surgical preparation of animals

As was described in the published studies, myocardial I/R injury in SD rats was established by ligation of the left anterior descending coronary artery (LAD) for 30 min and followed reperfusion for 2 h [5,19]. In brief, rats were anaesthetized with sodium pentobarbital (50 mg/kg, Merck) through intraperitoneal injection. To confirm the level of anesthesia, we tested pedal and palpebral reflexes throughout the entire experiment. After intubation, rats were acutely instrumented for the measurement of hemodynamics. The heating pad was used to maintain the rat body temperature at 37 ± 0.5°C. A left thoracic incision was made in the fourth intercostal space, and the pericardium was open to expose the heart. A 6–0 silk suture slipknot was placed around the proximal LAD, successful ischemia was confirmed by epicardial cyanosis and marked arrhythmia, while successful reperfusion was confirmed by epicardial hyperemic. After 30 min of LAD occlusion, the slipknot was released, allowing the myocardium to be reperfused for 2 h. Hemodynamics was continuously detected by a polyethylene catheter. It was placed into the LV and connected to a pressure transducer for data collection acquisition. Hemodynamics and LV functions such as mean arterial blood pressure (MAP), heart rate (HR), and rate pressure product (RPP) were recorded at the end of equilibration (T_0_), 30 min (T_1_), 60 min (T_2_), 90 min (T_3_) and the end (T_4_) of reperfusion, respectively.

### Experimental Protocol

As was illustrated in S1 Fig, rats were randomly divided into four groups: (1) SHAM group: rats underwent the same operation, except that the suture was placed around LAD but not tied. (2) I/R group: rats were subjected to 30 min of LAD occlusion, followed by 2 h reperfusion. (3) SPC group: rats with myocardial ischemia received 1.0 minimum alveolar concentration (MAC) sevoflurane (2.4% sevoflurane, 37°C) for 15 min at the onset of reperfusion, followed by 105 min reperfusion without sevoflurane. (4) Sevoflurane alone (SEVO) group: rats received 1.0 MAC sevoflurane for 15 min without occlusion.

### Myocardial infarct size

Myocardial infarct size was evaluated by 2,3,5-triphenyltetrazolium chloride triazole (TTC) staining [5,20]. Rat hearts were removed rapidly at the end of reperfusion. After washed with phosphate buffer solution (PBS, pH 7.4) and frozen at -20°C for 2 h, the LV was cut into 6 pieces with cross-section of 2-mm thickness. The hearts were incubated in 1% TTC in 0.1 mol/L PBS at 37°C for 15 min respectively, and subsequently fixed in 10% formalin solution (pH 7.4) for 12 h. The infarct area (IS, white color) and area at risk (AAR, red color) were identified by using a dissecting microscope. The IS and AAR were calculated digitally using Alpha Ease FC Imaging System, and the IS was expressed as a percentage of the AAR (IS/AAR, *n* = 6 /group).

### Echocardiography

Two-dimensional echocardiography was performed in unconscious (sodium pentobarbital 50 mg/kg, i.p.) rats using the Vevo770 system equipped with a 17.5-MHz linear transducer after 2 h reperfusion. The independent professional echocardiographer was blinded to the grouping. All parameters of cardiac function were evaluated by M-mode tracings at the papillary muscle level and averaged using ≥ 5 cardiac cycles by the same observer in a blinded manner (*n* = 10 /group).

### Histology and immunofluorescence

At the end of reperfusion, the hearts tissue was immediately collected for paraffin section and cryosectioning. The staining of hematoxylin-eosin (H&E) was performed on 3–5 μm sections of cardiac tissue cut from the 10% formaldehyde solution-fixed, paraffin-embedded blocks [21] (*n* = 3 /group). Immunofluorescence for Vimentin was performed on cryosectioning. After blocking with 3.5% normal goat serum, the cryosections with anti-Vimentin antibody were incubated overnight at 4°C. Subsequently, the sections were washed three times with PBS and incubated with Cy3-conjugated second antibody for 1 h at room temperature. At last, the sections were washed, counterstained for nuclei localization with Hoechst 33342. The H&E staining and fluorescence staining were observed with a light or confocal microscope (Zeiss Ltd., Germany). Vimentin immunofluorescence images were analyzed by using the Image J software (*n* = 3 /group).

### Transmission electron microscope

Hearts were rapidly removed at the end of reperfusion and LV tissues were cut into ultrathin sections (50–80 nm) and fixed with an ultramicrotome (Ultracut E, Leica). The sections were collected on 200 mesh copper grids (Ernest F. Fullam, Inc.) and contrast-stained with 1% uranylacetate. Sections were examined with transmission electron microscope by an independent investigator through using H-600 electron microscope (Hitachi Limited, Tokyo, Japan, *n* = 3 /group).

### Measurement of ATP

The level of myocardial ATP was measured by using a bioluminescence method as was previously described [22]. Briefly, at the end of reperfusion, the myocardial tissue was rapidly frozen in liquid nitrogen. In order to determine myocardial ATP concentration, the myocardium samples was removed from the liquid nitrogen, kept in the same proportion of ATP assay buffer, and homogenized with 50 mmol/L Tris-acetate buffer containing 2 mmol/L EDTA (pH 7.75), 1% NP-40, 150 mmol/L NaCl, and 0.1% SDS. The homogenized tissue was centrifugated at 12,000 × g for 30 min to pellet insoluble materials. Then, the supernatant added to a 96-well plate for ATP assessment. According to the manufacturer's instructions, myocardial ATP levels were assessed with ATP assay system (Jiancheng BioTech, Nanjing, China) and ATP standards for ATP quantification. For ATP content measurement, six hearts were assessed in different experimental groups (*n* = 6 /group).

### Oxidation parameters

To evaluate tissue production of ROS, fresh and frozen LV myocardium (10 μm sections) was incubated with 10 μmol/L DHE in PBS in the dark for 30 min at room temperature [4,23]. Followed by three washes in PBS, the sections were mounted with prolong gold antifade reagent and coverslipped. Tissue slides were observed with a laser scanning confocal microscope (Zeiss Ltd., Germany). The level of malondialdehyde (MDA) was examined using assay kits according to the manufacturer’s instructions. Total reduced glutathione (GSH) and oxidized glutathione (GSSG) were assayed by the method of Griffith [24] and using 5,5'-dithiobis(2-nitrobenzoic acid)(DTNB)-GSSG reductase recycling assay kit (*n* = 6 /group).

### Real time-PCR

For reverse transcription, total RNA from cardiac tissues was extracted using TRIzol (Invitrogen, Carlsbad, CA. USA) according to the instruction in the manufacturer. Two micrograms of RNA was reverse-transcribed into first strand complementary DNA (cDNA) synthesis by using the oligo (dT) first strand primer. After cDNA synthesis, the mRNA expression levels of Cycs, Cox4i1, Ndufa2, Ndufa4, Ndufa8, Cox7a1, Cox7a2 and TFAM were determined by real-time PCR using the FastStart Universal SYBR Green Master (Roche, Indianapolis, USA). The β-actin was used as an internal control, the primers are listed in supplemental S1 Table (*n* = 6 /group).

### Immunoblot analysis

Immunoblot was performed as we described previously [5]. In brief, cellular protein was extracted from cardiac LV tissue. After boiled for 5 min at 100°C with 5× loading buffer, equivalent amount of protein preparations (30–60 μg) were separated on 10% SDS-polyacrylamide gel electrophoresis (SDS-PAGE), transferred onto Immobilon-P membranes (Millipore, Bedford, Mass) and blocked by 5% non-fat milk. Then, the membranes were probed with appropriate primary antibodies overnight at 4°C followed by incubation with peroxidase conjugated secondary antibodies. The signals were detected by enhanced pierce chemiluminescence and visualized on X-ray films. H3 and Tubulin served as loading control, nuclear protein was expressed as ratios of normalized to H3 and cytoplasmic protein was expressed as ratios of normalized to Tubulin (*n* = 4 /group).

### Immunoprecipitation

To determine the interaction of Vps34 and Beclin1, immunoprecipitation was performed. Protein extracts from cardiac tissues were incubated with primary anti-Beclin1 antibody or anti-Vps34 antibody overnight at 4°C with gentle rotation, and subsequently incubated with protein A/G agarose slurry. After five times of thoroughly washing with the lysis buffer, the samples were mixed up with SDS-PAGE sample buffer, and then the mixture was heated 10 minutes and centrifuged.

The supernatants containing Beclin1 or Vps34 immunoprecipitates were removed and subjected to Western blot, in order to detect the mutual effect of Vps34 and Beclin1.

### Statistical analysis

The data are expressed as means ± standard deviation ($\bar{x}$± SD). One-way ANOVA was used for comparisons. Post-hoc procedure (Tukey’s test) was performed for multiple-range tests. *P* < 0.05 was considered to be statistical significance.

## Results

### SPC promotes hemodynamic performance after I/R

Following the experimental protocol, ninety-eight rats were used in this study. The mortality among three groups was not significantly different (0.0%, 0/31, 8.8%, 3/34, 6.1%, 2/33, respectively, *P* > 0.05).

To determine the protective role of SPC on myocardial I/R injury, we evaluated hemodynamics in rats treated with or without SPC. At baseline (T_0_) there were no statistical difference of the parameters measured (Table 1). During the occlusion and reperfusion of LAD, the HR, MAP and RPP in I/R and SPC groups decreased significantly (*P* < 0.05 vs baseline). Compared with the SHAM group, the values of HR, MAP and RPP were lower in the I/R group (*P* < 0.05). After 2 h reperfusion, the hemodynamic defect in rats with SPC treatment was attenuated, while the rats in I/R group were not (*P* < 0.05, Table 1).

**Table 1:** Myocardial hemodynamics during *in vivo* experiments.

Group	Baseline	Reperfusion			
	(T_0_)	30 min (T_1_)	60 min (T_2_)	90 min (T_3_)	2 h (T_4_)
HR(bpm)					
SHAM	320 ± 15.7	315 ± 12.8	314 ± 5.7	308 ± 6.3	303 ± 10.7
I/R	311 ± 12.7	291 ± 8.2	237 ± 5.7* ^#^	172 ± 8.4* ^#^	132 ± 6.1* ^#^
SPC	311 ± 5.6	278 ± 14.1	251 ± 5.2* ^#^	212 ± 8.0* ^#^ ^&^	185 ± 11.3* ^#^ ^&^
MAP(mmHg)					
SHAM	123 ± 3.5	122 ± 9.2	116 ± 8.2	114 ± 4.7	112 ± 6.5
I/R	114 ± 5.3	97 ± 6.3	85 ± 5.5* ^#^	59 ± 4.9* ^#^	42 ± 8.0* ^#^
SPC	119 ± 3.9	122 ± 9.2	99 ± 6.8* ^#^ ^&^	93 ± 5.5* ^#^ ^&^	83 ± 4.3* ^#^ ^&^
RPP(min^-1^ mmHg×10^3^)					
SHAM	46.1 ± 3.7	44.8 ± 6.1	43.0 ± 3.7	41.1 ± 3.7	40.6 ± 4.1
I/R	46.7 ± 2.2	40.4 ± 1.5	26.7 ± 1.7* ^#^	20.0 ± 2.2* ^#^	12.9 ± 1.8* ^#^
SPC	47.8 ± 1.6	44.3 ± 4.0	36.9 ± 3.8^&^	27.9 ± 2.3* ^#^ ^&^	21.9 ± 2.7* ^#^ ^&^

* *P* < 0.05 vs. T_0_;

^#^ *P* < 0.05 vs. SHAM group;

^&^ *P* < 0.05 vs. I/R group.

Values are means ± SD (*n* = 6 /group). HR, heart rate; MAP, mean arterial blood pressure; RPP, rate pressure product.

### SPC reduces myocardial infarction size, improves postischemic cardiac dysfunction and alleviates histopathological changes

**Fig 1:**
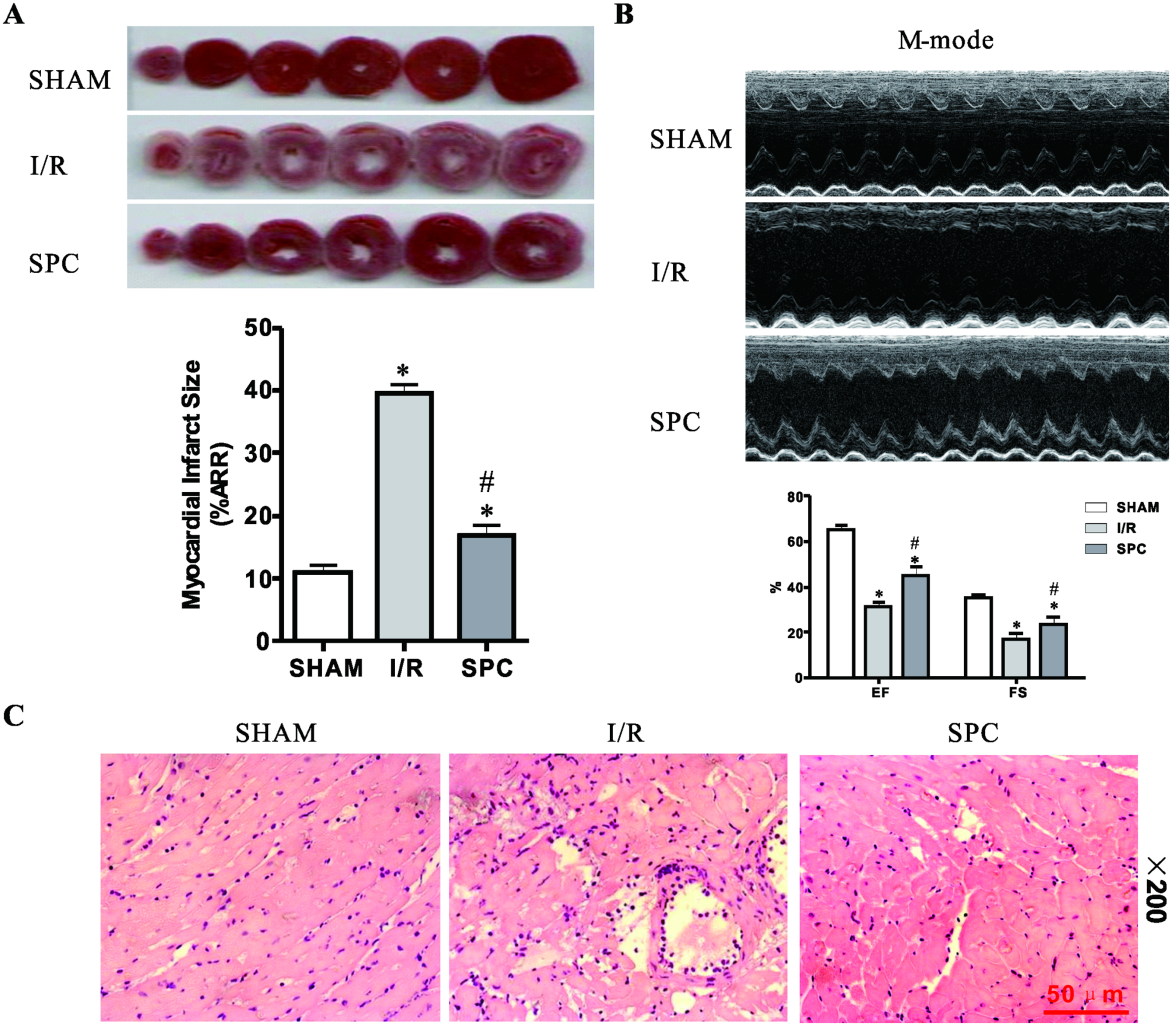
SPC decreases cardiac infarct size, increases LV contractile function and attenuates histopathologic organ damage following I/R. **(A)** Rats were sacrificed at the end of reperfusion and the hearts removed and stained with TTC for the measurement of myocardial infarct area. The infarct size was expressed as a percentage of area at risk. *n* = 6 /group. **(B)** Echocardiography was performed at the end of reperfusion and representative M-mode echocardiograms were recorded in all groups. Rats without LAD occlusion served as basal controls (SHAM group). *n* = 10 /group. **(C)** LV tissues were retrieved at the end of reperfusion, paraffin section was prepared and subjected to the H&E staining. Representative H&E staining images are shown (magnification, 200×). Scale bar: 50μm. *n* = 3 /group. The columns and errors bars represent means ± SD. * *P* < 0.05 vs. SHAM group; # *P* < 0.05 vs. I/R group.

To further confirm that SPC has cardioprotection against I/R injury, myocardial infarction size was measured and cardiac function was assessed using echocardiography. In line with the results of previous study [25], our data demonstrated that LAD occlusion induced significant myocardial infarction in the I/R group (39.57±3.47% of the total area, (Fig 1A). Administration of 1.0 MAC sevoflurane at the beginning of reperfusion for 15 min substantially reduced the infarct size to 17.13±4.19%, which was significantly smaller than the infarction area in I/R rats without any treatment (*P* < 0.05). We then examined LV systolic function by echocardiography. LV contractility was reflected by EF%, FS%, SV, LVIDd and LVIDs. Due to loss of viable myocardium, cardiac systolic dysfunction was observed in I/R and SPC groups (Fig 1B and 1C). Compared with the SHAM group, EF% and FS% were profoundly reduced in I/R group (31.47±1.71% vs. 64.89±1.91%; 16.83±2.60% vs. 34.98±1.45%, respectively, *P* < 0.05, S2 Table). LVIDd and LVIDs were also significantly increased (*P* < 0.05), which indicated a decrease in the LV contractility. In contrast, the administration of SPC improved LV systolic function against I/R injury. As was shown in S2 Table, the EF%, FS% and SV significantly increased in the SPC group when compared with those in the I/R group (*P* < 0.05). SPC reduced I/R-induced cardiac dilation as measured by LVIDs and LVIDd (5.25±0.32 vs. 6.32±0.74; 6.74±0.44 vs. 7.69±0.69, respectively, *P* < 0.05). Noteworthy, indicators of cardiac remodeling (IVSd, LVVs, LVVd) were also improved in the SPC group (*P* < 0.05 vs. I/R group, S2 Table). Semiquantitative analysis of myocardial I/R injury was conducted in H&E-stained heart sections [26]. As shown in Fig 1C, the myocardial structure in the SHAM group was arranged regularly, and cardiomyocytes presented with a normal size, clear boundaries and arranged regularly. Compared with the SHAM group, myocardial fibers presented aberrantly and in a wavy arrangement, myocardial fibers were irregular and difficult to recognize their outlines in certain areas, and transverse striation was unclear or disordered in I/R group. In addition, the cardiomyocytes appears edema with neutrophil infiltration and large necrotic area were observed in the heart of rats subjected to I/R injury. The injury of the myocardium in the SPC group was significantly relieved when compared with the rats in I/R group. In the SPC group, we observed that widening of cardiac muscle fibers lined up in order, the transverse striation and the integrity of cardiomyocytes were clear and well-distributed (Fig 1C).

Taken together, these results demonstrate that SPC has a protective effect against myocardial I/R injury in rat model.

### SPC preserves the ultrastructural integrity, increases myocardial ATP content, inhibits the mPTP opening and improves mitochondrial dysfunction

**Fig 2:**
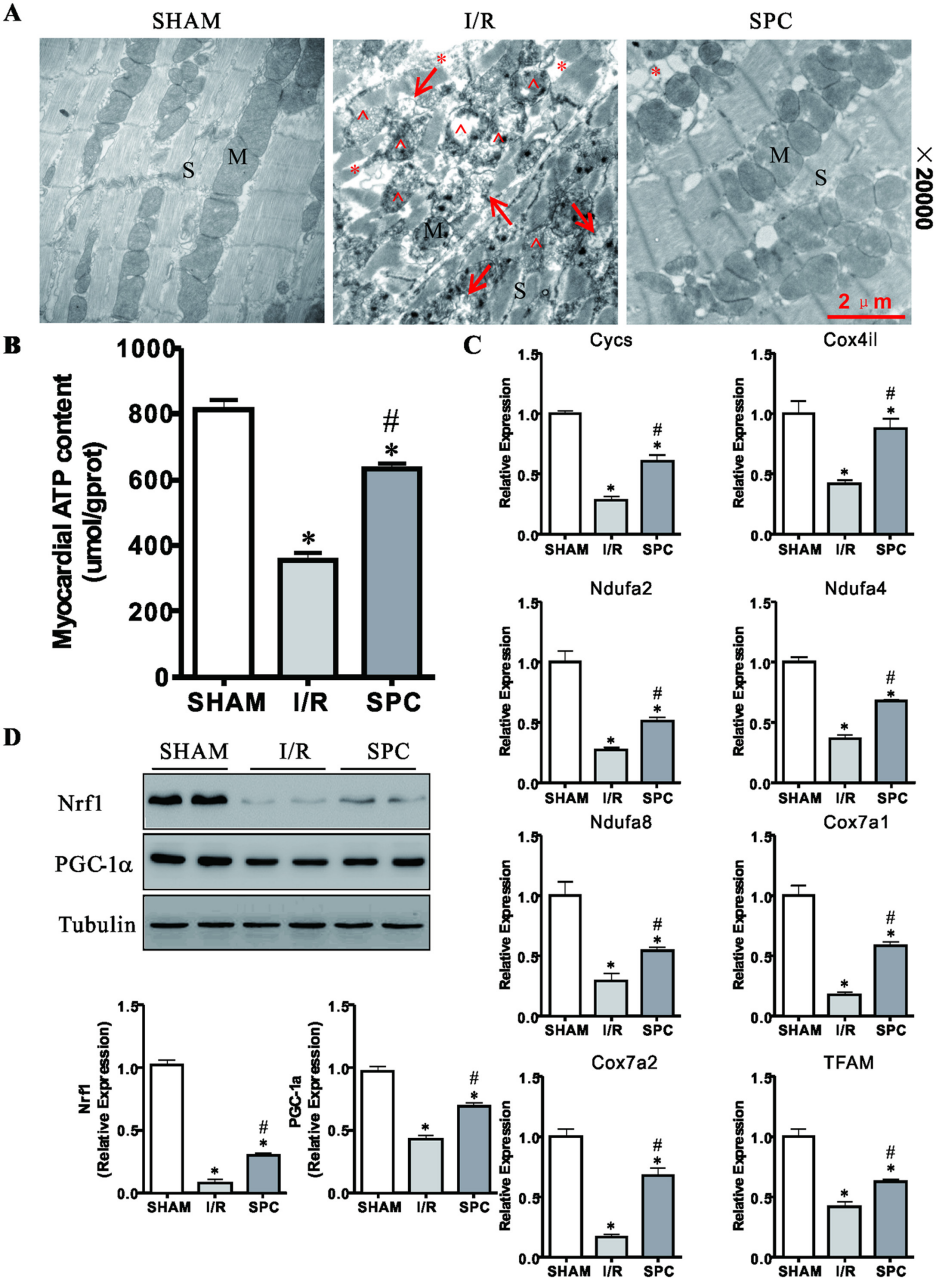
SPC ameliorates mitochondrial disorder and dysfunction after I/R. **(A)** LV tissues were harvested for examination of myocardial ultrastructure by transmission electron microscopy (TEM). Typical TEM images obtained at a magnification (20000×) of cardiac ultrastructure in all groups. Note that myofilaments were absent (*) and damaged mitochondria (^) nearby the autophagosomes (→). Scale bar: 2μm. *n* = 3 /group. M: mitochondria; S: sarcomeres. **(B)** SPC prevents depletion of ATP stores in I/R hearts and the ATP content in all groups are shown. *n* = 6 /group. **(C)** After 2 h of reperfusion, LV tissues were obtained and analyzed by real-time PCR for the expression levels of Cycs, Cox4il, Ndufa2, Ndufa4, Ndufa8, Cox7a1, Cox7a2 and TFAM. *n* = 6 /group. **(D)** LV were collected and prepared for immunoblots. Representative immunoblots and semiquantitative analysis of Nrf1 and PGC-1 in each group of rats after reperfusion. The blots for Tubulin were served as loading controls. *n* = 4 /group. All data are presented as means ± SD. * *P* < 0.05 vs. SHAM group; # *P* < 0.05 vs. I/R group.

To test whether SPC improved cardiac ultrastructural damages, we examined myocardial ultrastructure by transmission electron microscope (TEM). Fig 2A illustrated ultrastructural sections of SHAM group, showed well-arranged sarcomeres, normal myofibrils, mitochondrial structure with intact cristae density and uniformly scattered glycogen granules. However, I/R challenge resulted in marked ultrastructural damages, which was evidenced by myofibrillar derangement and extensive sarcomeres absent. Additionally, swollen and irregular mitochondria with a more pronounced vacuolation and cristae disruption were observed (Fig 2A). Electron photomicrographs revealed well-arranged sarcomeres, normal myofilaments ultrastructure and relatively normal structure of mitochondria without vacuolation and cristae disappear in sevoflurane treated group. However, mild rarefaction of myofilaments was also observed.

Energy depletion leads to LV contractile insufficiency [27]. The loss of ATP content, mitochondrial morphological abnormalities, and broken energy transfer system are well known markers for energy depletion [28]. Mitochondria occupy one third of the cell volume in mammalian cardiomyocyte and supply 95% ATP for the hearts [29]. As cellular ATP is produced in the mitochondria, and mitochondrial dysfunction were observed in I/R group; therefore, myocardial ATP was measured. In line with the TEM results, the myocardial ATP level in I/R group was significantly lower than that in SHAM group (810.62±74.57 vs. 354.71±51.20 umol/gprot, *P* < 0.05, Fig 2B). And compared with rats in the I/R group, the ATP content in SPC group increased by 78.3% (*P* < 0.05). We next assessed the transcriptional levels of mitochondrial function-related genes. Compared with the rats in the SHAM group, the mRNA levels of Cycs, Cox4il, Ndufa2, Ndufa4, Ndufa8, Cox7a1, Cox7a2 and TFAM were decreased in the I/R and SPC groups (*P* < 0.05, Fig 2C). And compared with the rats in I/R group, the levels of these mRNA in SPC group increased by 118.1%, 107.0%, 88.3%, 72.1%, 82.2%, 221.3%, 98.1% and 48.8%, respectively (*P* < 0.05). Moreover, as shown in Fig 2D, the expression levels of mitochondria biogenesis regulator PGC-1α and Nrf-1 proteins were elevated in the SPC group when compared with the rats in the I/R group (*P* < 0.05), possibly as a compensatory response to mitochondrial dysfunction. Moreover, previous study has reported that pre-ischemic induction of autophagy restores sevoflurane preconditioning lost by longer ischemic insult. And, this effect is linked to enhanced inhibition of mitochondrial permeability transition pore (mPTP) by autophagy [30]. In order to further understand the effects of SPC on mitochondria, we evaluated the nicotinamide adenine dinucleotide nad (NAD^+^) contents in hearts among the three groups in this study. S2 Fig shows that SPC retained higher contents of NAD^+^ than that in the I/R group, which suggested that SPC prevented NAD^+^ release by inhibiting the mPTP opening.

To sum up, these results suggest that SPC effectively maintained the normal mitochondrial structure and function.

### SPC inhibits myocardial oxidative stress

**Fig 3:**
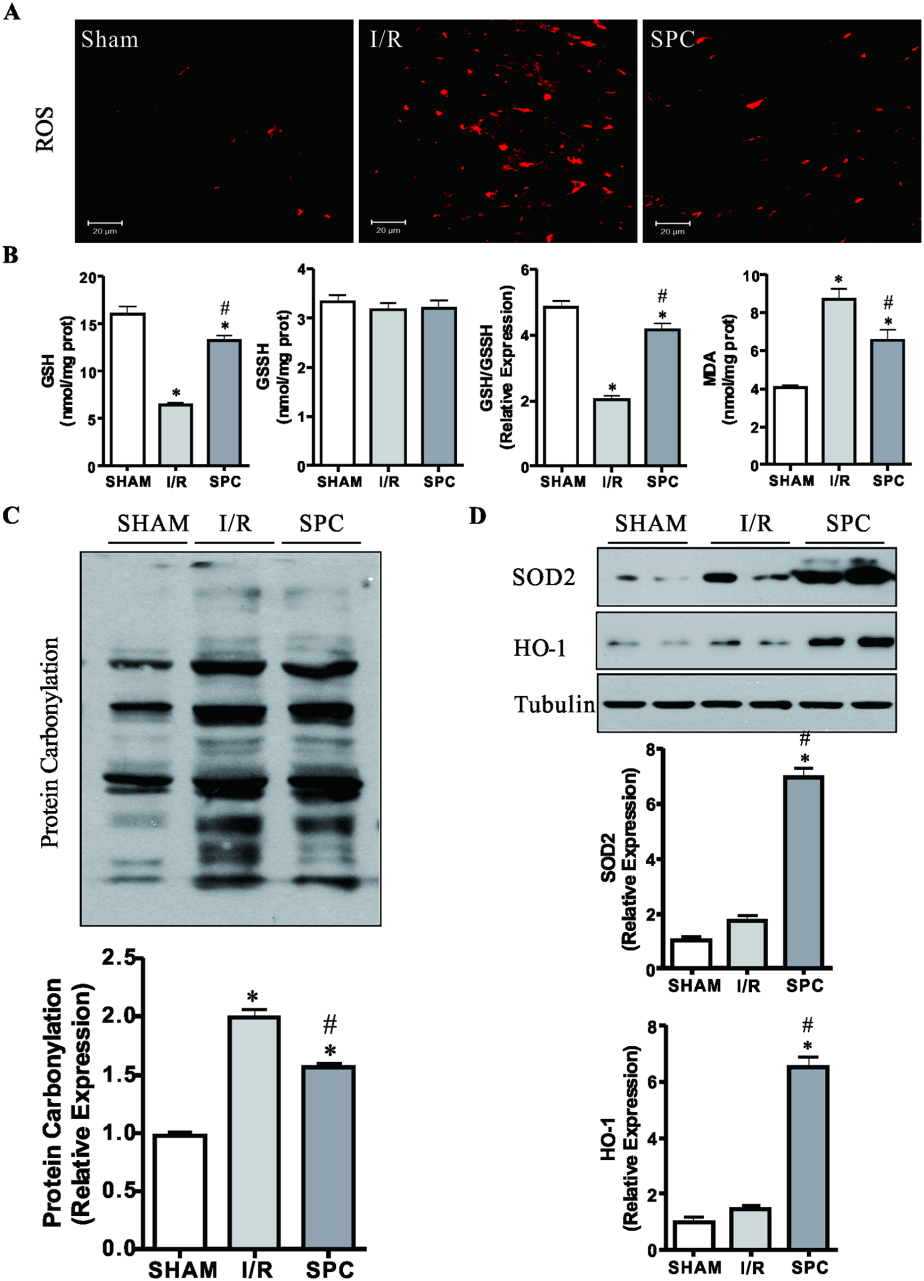
SPC inhibits oxidative stress brought about by the I/R injury. **(A)** The cardiac levels of ROS in each group are shown by DHE staining. The image was obtained by a confocal microscope. SPC-treatment significantly lowered the increased DHE fluorescent intensity induced by the I/R injury. Scale bar: 20μm. *n* = 3 /group. **(B)** The cardiac GSH, GSSG and MDA levels were measured using enzymatic kits, and the GSH/GSSG ratios derived from the GSSG and GSH contents. *n* = 6 /group. **(C)** Protein carbonyl content was examined as carbonyl-containing 2,4-DNPH adducts by immunoblotting. Protein carbonylation of SPC group was likewise lower than the I/R group. *n* = 4 /group. **(D)** The immunoblot analysis SOD2 and HO-1 expression at the end of reperfusion. The blots for Tubulin were served as loading controls. *n* = 4 /group. The columns and errors bars represent means ± SD. * *P* < 0.05 vs. SHAM group; # *P* < 0.05 vs. I/R group.

Oxidative stress plays a critical role in the development of myocardial I/R injury. Damaged and dysregulated mitochondria generate redundant amounts of ROS which leads to myocardial damage [31]. To investigate the relationship between the SPC-mediated cardioprotection and oxidative stress, we measured the cardiac levels of ROS, MDA, GSH, and GSSG among the rats in all groups. The cardiac levels of ROS and MDA in I/R group were higher than those in SHAM and SPC groups (*P<*0.05, Fig 3A and 3B). And I/R significantly decreased the GSH and the ratio of GSH/GSSG compared with the remaining two groups (*P* < 0.05, Fig 3B). Surprisingly, SPC treatment significantly decreased the contents of ROS and MDA, and raised the level of GSH and the ratio of GSH/GSSG (*P* < 0.05, vs. I/R group, Fig 3A and 3B). Protein carbonyl content of SPC group was also lower than that of I/R group (*P* < 0.05, Fig 3C). To further address antioxidative effect of SPC, we observed the expressions of SOD2 and HO-1 in all rats. As shown in Fig 3D, compared with the SHAM and I/R groups, SPC significantly increased the expressions of SOD2 and HO-1 (*P* < 0.05). These data indicate that SPC ameliorates oxidative stress via up-regulating the expression levels of SOD2 and HO-1 following I/R challenge.

### SPC decreases accumulation of protein aggregates

**Fig 4:**
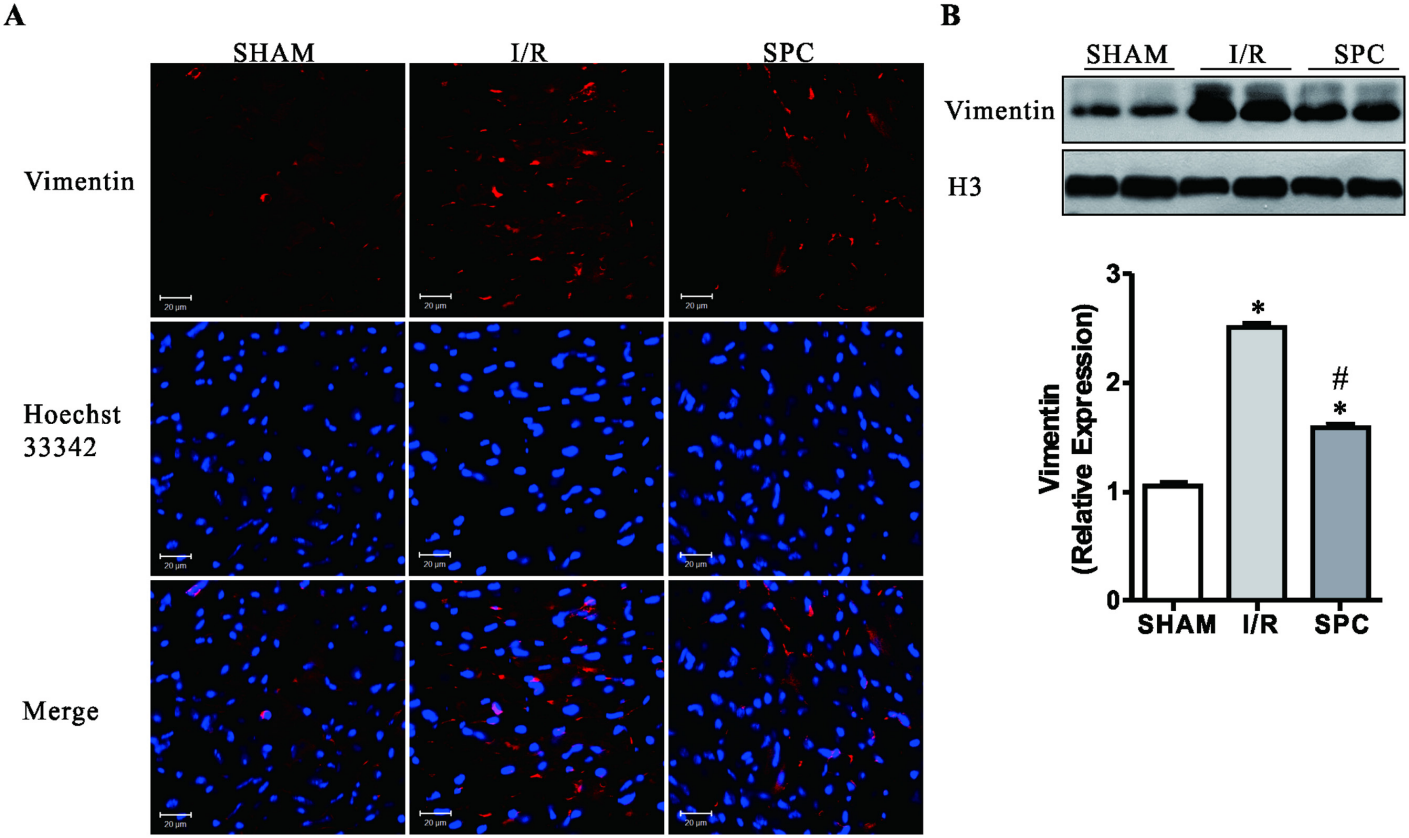
SPC narrows the I/R-induced accumulation of protein aggregates. **(A)** Cardiac tissues at papillary muscles level were collected and the cryosectioning was prepared. The immunohistochemical analysis of protein aggregates stained with anti-Vimentin is shown. Hoechst 33342 was used to stain cell nuclei. The immunofluorescence was examined by a confocal microscope. Scale bar: 20μm. *n* = 3 /group **(B)** Representative immunoblots of nuclear protein from all groups detected with a Vimentin-specific antibody. H3 was utilized to normalize the amount of protein. *n* = 4 /group. The columns and errors bars represent means ± SD. * *P* < 0.05 vs. SHAM group; # *P* < 0.05 vs. I/R group.

Aberrant protein aggregation has been found in many diseases, including dilated cardiomyopathy and ischemic heart disease [32]. Therefore, we assessed the levels of protein aggregation among all groups by immunostaining for Vimentin, a structural component of the aggresome [13]. Here, we detected a dramatic accumulation of Vimentin staining in I/R group when compared with SHAM controls (Fig 4A). Consistently, the expression level of Vimentin in I/R hearts significantly increased by 153% (*P* < 0.05 vs. SHAM group, Fig 4B). In addition, compared with the I/R group, SPC significantly decreased the expression of myocardial Vimentin (Fig 4A and 4B). Therefore, we concluded that **S**PC decreases superabundant protein aggregation.

### SPC increases the levels of p-AKT and p-mTOR in the myocardium following I/R

**Fig 5:**
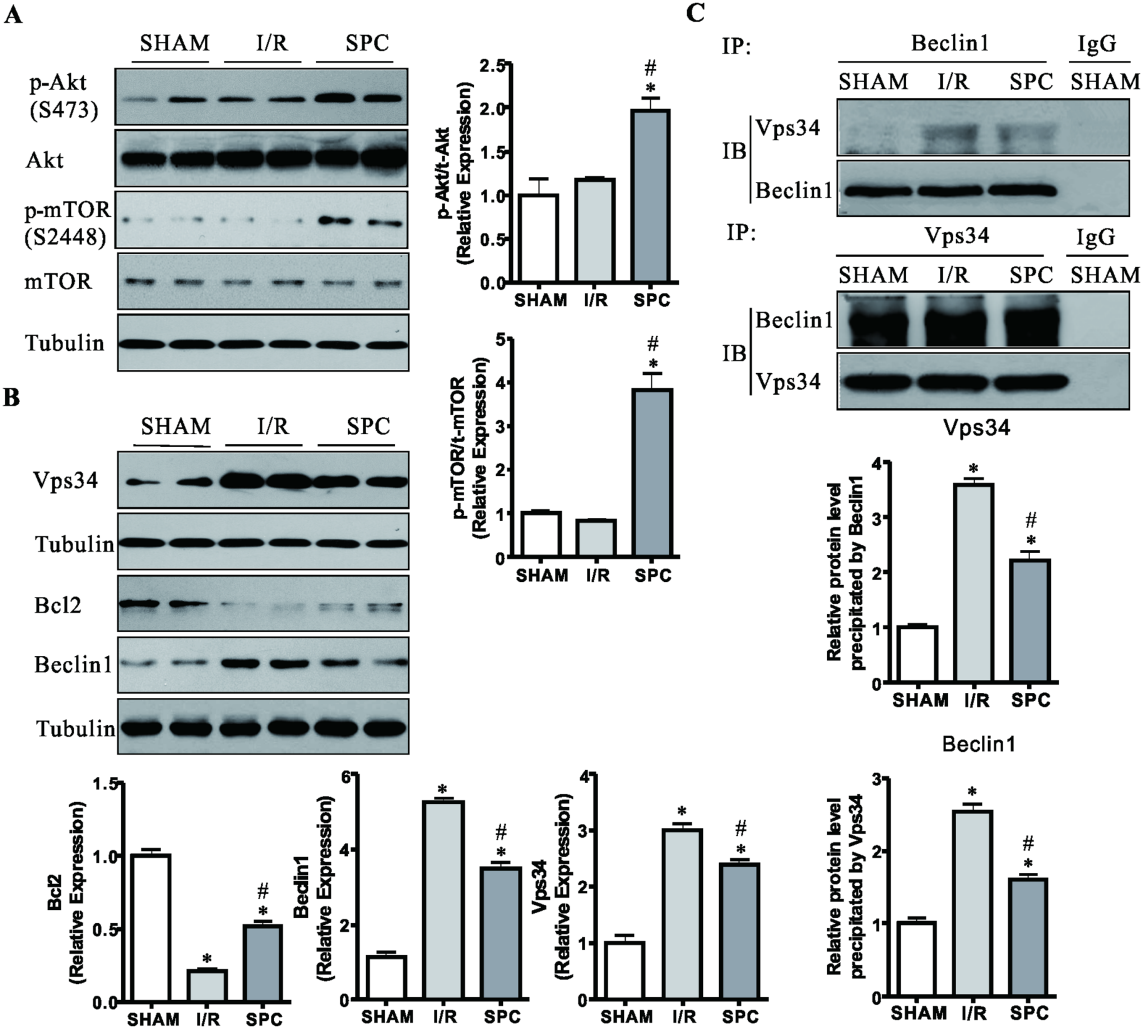
SPC plays an important regulatory role in the expression of class I/III PI3K and the interaction among Bcl2, Beclin1 and Vps34. **(A)** LV tissues were collected from rats at the end of 2h reperfusion. The immunoblotting for phosphor-Akt (Ser473), Akt, phosphor- mTOR (Ser2448) and mTOR were performed. *n* = 4 /group. **(B)** Immunoblotting was used to examine the expression levels of Vps34, Bcl2 and Beclin1. Representative protein images and quantitative analysis were shown. The blots for Tubulin were served as loading controls. *n* = 4 /group. **(C)** Effect of SPC on I/R-induced association between beclin1 and Vps34. *Upper panel*, Cell lysates from LV tissues were immunoprecipitated (IP) with Beclin1 antibody and immunoblotted (IB) for Vps34 and Beclin1; *Lower panel*, Lysates were immunoprecipitated with Vps34 antibody and immunoblotted for Vps34 and Beclin1. The columns and errors bars represent means ± SD. * *P* < 0.05 vs. SHAM group; # *P* < 0.05 vs. I/R group.

The PI3K plays an important role in signaling acute myocardial I/R injury and regulating mitochondrial function [33, 34]. We therefore examined the expression of class I PI3K (indicted by phosphorylation of Akt and mTOR). As shown in Fig 5A, the levels of phosphor-Akt (Ser473) and phosphor-mTOR (Ser2448) were comparable between SHAM and I/R groups. The phosphor-Akt (Ser473) and phosphor-mTOR (Ser2448) expression levels in heart tissue significantly increased in SPC group than the above-mentioned two groups (*P<*0.05, Fig 5A). The datum indicates that SPC protects cardiomyocytes from I/R injury by activating the class I PI3K/Akt/mTOR pathway.

### SPC results in downregulation of class III PI3K Vps34 and reduces I/R induced interaction of Vps34 with Beclin1

Activation of class III PI3K Vps34 contributes to autophagy activation [15]. We examined expression levels of Vps34 in myocardial tissues from all three groups. As shown in Fig 5B, Vps34 level in I/R group was significantly increased by 260.4% at the end of reperfusion, when compared with SHAM group (*P<*0.05). SPC decreased Vps34 level when compared with I/R alone.

In mammals, Bcl2 acts as an inhibitor of autophagy that inhibits the interaction between Vps34 and Beclin1. The formation of Beclin1/Vps34 complex is required for induction of class III PI3K activity and activation of autophagy [35]. Fig 5B shows that the levels of Bcl2 in the myocardium of SPC-treated rats were markedly increased by 143.4% compared with I/R rats. We then examined changes in the interaction of Vps34 with Beclin1 by immunoblotting of Beclin1 and Vps34 immunoprecipitates with antibodies directed against Vps34 and Beclin1, respectively. Compared with the SHAM group, the association between Beclin1 and Vps34 was dramatically increased by 266.5% in the myocardium of I/R group (*P<*0.05, Fig 5C). Combined with Fig 5B and 5C, SPC increased the level of Bcl2 protein, which reduced release of Beclin1 from the Bcl2/Beclin1 complex, and thereby significantly reduced the I/R induced formation of the Beclin1/Vps34 complex (*P<*0.05, Fig 5C).

Together, these data indicate that the SPC-induced upregulation of Bcl2 may reduce the interaction between Vps34 and Beclin1, resulting in inactivation of Vps34 which in turn to inhibit autophagy activity in I/R hearts.

### SPC suppresses the activation of mitophagy following I/R

**Fig 6:**
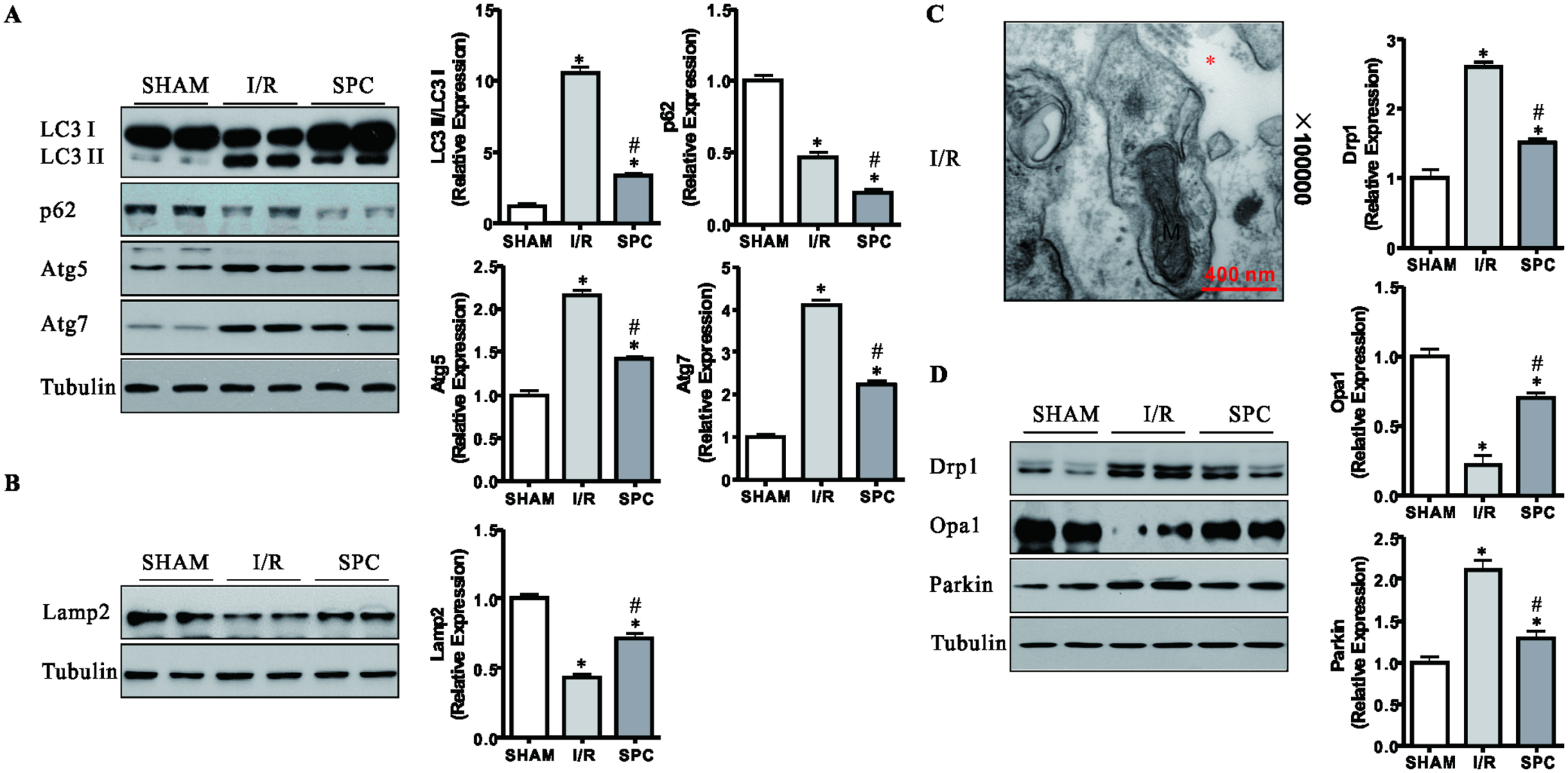
SPC can protect against I/R-induced mitochondrial fragmentation and ensuing mitophagy. **(A)** The expression levels of LC3 II/I, p62, Atg 5 and Atg 7 are pivotal markers of autophagosome formation and clearance. Representative immunoblot for these proteins in the myocardial tissues from all groups and densitometric quantification. *n* = 4 /group. **(B)** The autophagosome clearance related Lamp 2 was found out. Representative protein bands and densitometric quantification were shown. Tubulin was used to an internal control. *n* = 4 per group. **(C)** Representative electron micrograph of the heart from the I/R group obtained at a magnification of 100000 times. Asterisk (*) indicates dissolved myofilaments. Scale bar: 400nm. *n* = 3 /group. **(D)** Immunoblotting was used to examine the levels of Drp1, Opa1 and Parkin. Representative protein images and quantitative analysis were shown. Expression was normalized to Tubulin for each sample. *n* = 4 /group. The columns and errors bars represent means ± SD. * *P* < 0.05 vs. SHAM group; # *P* < 0.05 vs. I/R group.

Energy depletion, oxidative stress, protein aggregation and the formation of the Beclin1/Vps34 complex are important triggers of cardiomyocyte autophagy. The class III PI3K facilitates autophagosome biogenesis and maturation, and the class I PI3K reduces autophagic activity [13–15,36,37]. The above data indicate that SPC might protect the myocardium from I/R injury via the suppression of excessive autophagic activation in rat. To validate this hypothesis, we detected autophagosome formation related proteins (the LC3 conversion, Beclin1, Atg5 and Atg7) and the well-known markers for autophagosome clearance (p62 and Lamp2) [38]. Compared with the SHAM controls, I/R significantly increased the expressions of Beclin1, Atg5, Atg7 and the LC3 II/LC3 I ratio, indicating increased autophagosome formation (all *P* < 0.05, Figs 5B and 6A). In contrast, the level of p62 was markedly reduced by 53.4% and 77.9% in hearts in the group of I/R and SPC, respectively (*P* < 0.05 vs. SHAM group, Fig 6A). Meanwhile, I/R decreased cardiac Lamp2 level by 56.8% (*P* < 0.05 vs. SHAM group, Fig 6B). After SPC treatment, the increases of Beclin1, Atg5, Atg7 and the LC3 II/LC3 I ratio, and the reduction of Lamp2 were blunted, when compared with these in the I/R group, indicating SPC corrected excessive autophagy (all *P* < 0.05, Figs 5B, 6A and 6B). In addition, the p62 level was significantly decreased in SPC compared with I/R, indicating efficient autophagosome clearance in SPC rats.

Consistently, TEM images illustrated that SPC diminished the number of autophagosomes, when compared with the I/R group (Fig 2A). Interestingly, the TEM image of I/R group showed the autophagosome containing mitochondria and missing of myofilaments (Fig 6C). To further evaluate the effect of SPC on mitochondrial dynamics and clarify the types of autophagy, we detected the expressions of Drp1, Opa1 and Parkin. Activated Drp1 and reduced Opa1 caused mitochondrial fragmentation, an early step in mitophagy [39]. Then the interaction between Parkin and degradation damaged mitochondria [18]. As shown in Fig 6D, Opa1 protein expression in I/R group was decreased by 78.4% compared with SHAM group (*P<*0.05). In addition, the Drp1 and Parkin in I/R group were uniformly higher than SHAM group (*P* < 0.05, Fig 6D), suggesting the I/R injury enhanced myocardial mitophagy. Conversely, SPC significantly attenuated the decrease in Opa1 and increases in Drp1 and Parkin, respectively (all *P* < 0.05 vs. I/R group, Fig 6D). S3 Fig shows that there were no significant differences in autophagy related proteins (LC3, p62, Beclin1, Atg5 and Atg7) between sham operation rats and sevoflurane alone rats. Furthermore, the expression levels of the Drp1, Opa1 and Parkin proteins in hearts of SHAM group were comparable with that in gender-matched SEVO group (S4 Fig).

Taken together, these data imply that the inhibition of mitophagy is probably involved in the cardioprotective effect of SPC in cardiac I/R injury.

## Discussion

The findings of this study were to extend the understanding of SPC on the protection of myocardial I/R injury. First, SPC was proved to be effective for protection of the heart against the I/R injury *in vivo* rat model, as was shown by significantly attenuated cardiac function, decreased area of myocardial infarction and reduced histological damage after I/R injury. In addition, SPC-induced cardioprotection was realized by improvement of mitochondrial energy metabolism, prevention of oxidative stress, amelioration of intracellular protein aggregation and regulation of the PI3K-related signaling molecules.

The volatile anesthetic sevoflurane was synthesized in 1975 and used in clinical practice over 20 years. Sevoflurane not only produces excellent anesthesia effectiveness with little systemic toxicity, but also plays an important role in resistance to myocardial I/R injury. Almost a decade ago, 1.0 MAC of sevoflurane administered during the first 2 min of 2h reperfusion period was shown to protect against myocardial I/R injury in the rat *in vivo* [40]. Emerging evidence has demonstrated that postconditioning with sevoflurane was a potent strategy for protection of ischemic myocardium *in vivo and in vitro* [8–10,40]. Furthermore, the sevoflurane induced cardioprotective effects were influenced by many factors, such as gender. Recent evidence showed that SPC developed cardioprotection in male rats but not in female rats [41]. However, major molecular mechanisms underlying SPC-induced cardioprotection in male rats are still indistinct.

We observed that LV systolic dysfunction was exhibited in I/R group and cardiac function was improved by SPC. Myocardial I/R injury leads to mitochondrial dysfunction, which in turn to induce the increase of intracellular ROS production and Ca^2+^ level that is momentous factors in the development of contractile dysfunction [42]. Among the potential molecular mechanisms responsible for SPC-induced improvement of cardiac function, pathway signaling seems to be focussed on the structure and function of mitochondria [43,44]. We also observed prevention of mitochondrial destruction and increase of ATP content in SPC hearts. And, SPC administration significantly increased transcriptional levels of the genes related to mitochondrial function (Cycs, Cox4il, Ndufa2, Ndufa4, Ndufa8, Cox7a1, Cox7a2 and TFAM). Consistently, the expressions of mitochondrial proteins (Nrf-1, PGC-1α) were significant up-regulated after SPC administration. Our results indicate that the cardiac protection of SPC in remission of acute LV systolic dysfunction may involve the protection of mitochondria.

Mitochondria is one of the most active and important organelles in the cardiomyocyte, consuming almost 90% of the oxygen content to enable 95% of the hearts ATP synthesis and oxidative phosphorylation [45]. Mitochondria is both generators of and targets for ROS, the damaged mitochondria generate a lot of ROS which can cause oxidative stress during both ischemia and reperfusion [46,47]. Oxidative stress in turn aggravates the mitochondrial dysfunction and ultimately mediates cell death. However, the SOD2 (mitochondrial antioxidant marker) and HO-1 (antioxidant marker) proteins are known to play critical cardioprotective roles in I/R injury [48]. Our further study discovered that SPC could significantly reduce the excessive production of ROS and the elevated protein carbonylation, which then increased expressions of antioxidant protein SOD2 and HO-1. This observation indicates that SPC protected myocardium against I/R-induced oxidative stress by activating SOD2 and HO-1.

The cross-talk between redox signalling and mitochondrial dysfunction in I/R injury is not clearly understood. Some possibilities include the accumulation of protein aggregates and the deterioration of mitochondrial function, lead to the vicious cycle of further oxidative stress [49]. Indeed, we observed a significant increase in the accumulation of protein aggregates in the I/R group. In contrast, SPC has been shown to decrease the expression level of Vimentin, suggests that cardioprotective effect of SPC against myocardial I/R injury is via ameliorate protein misfolding and aggregation.

Under normal circumstance, autophagy has an important role for maintaining intracellular homeostasis and cardiomyocyte survival. Interestingly, it remains unclear that whether the autophagy activation is protective or harmful in the heart after I/R challenge. A recent study showed that autophagy activation clearly reduced the myocardial infarct size in a mouse model of I/R injury [16]. Mounting evidences now support that energy depletion, ROS and protein aggregation induce autophagy [13–15,36,37]. We found that energy depletion, oxidative stress and protein aggregation were exhibited in untreated I/R hearts. Moreover, we also observed increased number of autophagosomes, the up-regulated expression of LC3 II, Atg5, Atg7 and Beclin1 and decreased level of Lamp2 protein in I/R hearts, suggesting that autophagy was enhanced after I/R. Our study indicated that, in contrast to previous study, activation of autophagy was detrimental during I/R. In supporting our findings, I/R promotes the development and activity of autophagy and causes autophagic cell death in H9c2 cells [50]. Consistently, the previous study showed that autophagy activation can aggravate myocardial injury following I/R [51]. Using *in vivo* mice models, a current study demonstrated that I/R injury impairs autophagosome clearance by decline in Lamp2 and up-regulation of Beclin1. And blockade of autophagy flux leads to autophagosome accumulation and even cell death [52]. Coincidentally, our data further support this standpoint. What’s more, we found SPC-treatment could inhibit the activation of autophagy and restore the expression of Lamp2 levels accompanying with the decrease expression of Vps34 and the increase expressions of p-Akt (Ser473) and p-mTOR (Ser2448). The class I PI3K (indicated by p-Akt and p-mTOR) enzyme produce PI(3,4) P2 and PI(3,4,5) P3 that have been shown to decrease autophagic activity [53]. The only class III PI3K enzyme, Vps34 produces PI(3)P that is known to promote the formation and maturation of autophagosome [53]. Taken together, our findings suggest that SPC restores autophagosome processing and attenuates I/R-induced cardiomyocyte death by the activation of class I PI3K but not class III PI3K.

Intriguingly, the electron microscope examination exhibited that I/R-induced defective mitochondria were likely engulfed by autophagosome, also favoring mitochondrial autophagy or mitophagy. As an important subclass of autophagy, mitophagy plays a pivotal role in the regulation of mitochondrial homeostasis and dynamics in I/R stress [54]. The imbalance in mitochondrial dynamics [activation of dynamin-related protein-1 (Drp1) and suppression of optic atrophy 1 (Opa1)] promotes mitochondrial fragmentation which initiates mitophagy. Drp1 promotes mitochondrial fission, while Opa1 controls fusion [39,55]. In addition, Parkin, an E3 ubiquitin ligase localized in the cytoplasm, interact with activated Drp1. Parkin can recruite to the damaged mitochondrial outer-membrane and signal mitochondrial degradation under stress [17,18]. Our data confirmed that prominent mitophagic activity was associated with the significant up-regulation of Drp1 and Parkin and the down-regulation of Opa1 in I/R group. On the contrary, SPC inhibits the excessive activation of mitophagy. These findings suggest that high levels of mitophagic activity could be involved in the progress of acute I/R injury in rats. However, SPC-induced mild mitophagy as a pro-survival mechanism to eliminate damaged mitochondria and prevent the development of I/R injury.

In conclusion, our data demonstrated that SPC treatment effectively protected the rat heart from I/R injury *in vivo*. This action of SPC may involve restoration of mitochondria-related bioenergetic metabolism and autophagosome clearance, inhibition of excessive ROS production, amelioration of protein aggregation, activation of class I PI3K, down-regulation of the expression of class III PI3K and suppression of autophagy (mitophagy) at the end of reperfusion. In this context, the relationship among these mechanisms in SPC-induced cardioprotection is still unclear. Further intensive studies are needed in order to clarify the causal link between these mechanisms.

## Supporting Information

S1 Fig Experimental protocol. All the groups underwent the same surgical operation. (1) SHAM: rats were subjected to open chest surgery only; (2) I/R: rats were subjected to 30 min LAD occlusion, followed by 2 h of reperfusion; (3) SPC: rats were subjected to I/R and receiving 2.4% sevoflurane [1.0 minimum alveolar concentration (MAC) at 37°C] for 15 min at onset of reperfusion; (4) SEVO: rats received 1.0 MAC sevoflurane for 15 min without occlusion.
(JPG)

S2 Fig Myocardial NAD^+^ content after 15 min of reperfusion. Compared with the SHAM group, the NAD^+^ content in the other groups decreased. And, the NAD^+^ content in the SPC group was significantly higher than I/R group, SPC prevents reducing of myocardial NAD^+^ content after I/R. *n* = 6 /group. NAD, nicotinamide adenine dinucleotide.
(JPG)

S3 Fig Administration of sevoflurane without occlusion does not affect the expressions of autophagy associated proteins. At the end of reperfusion, the autophagy associated proteins were compared between SHAM and SEVO group. Expressions of LC3 II/LC3 I ratio, Beclin1, Atg5 and Atg7 in SHAM and SEVO groups were measured by western blot. The autophagy associated proteins were no significant difference between SHAM group and SEVO group (*P* > 0.05). *n* = 4 /group.
(JPG)

S4 Fig Administration of sevoflurane without occlusion does not affect the expressions of mitophagy associated proteins. At the end of reperfusion, the mitophagy associated proteins were compared between SHAM and SEVO group. The expressions of Drp1, Opa1 and Parkin in SHAM and SEVO groups were measured by western blot. The mitophagy associated proteins were no significant difference between SHAM group and SEVO group (*P* > 0.05). *n* = 4 /group.
(JPG)

S1 File Reagents and Measurement of mPTP opening. (DOC)

S1 Table Primers used in real-time PCR. Cycs, Cytochrome C; Cox4i1, Cytochrome c oxidase subunit 4 isoform 1; Ndufa2, NADH dehydrogenase (ubiquinone) 1 alpha subcomplex subunit 2; Ndufa4, NADH dehydrogenase (ubiquinone) 1 alpha subcomplex subunit 4; Ndufa8, NADH dehydrogenase (ubiquinone) 1 alpha subcomplex subunit 8; Cox7a1, Cytochrome c oxidase subunit VIIa polypeptide 1; Cox7a2, Cytochrome c oxidase subunit VIIa polypeptide 2; TFAM, transcription factor A mitochondrial.
(DOC)

S2 Table Echocardiographic measurements 2 h after IR. * P <0.05 vs SHAM group; # *P* <0.05 vs I/R group. LVIDs, left ventricular internal diameter at systolic phase; LVIDd, left ventricular internal diameter at diastolic phase; IVSs, interventricular septal thickness at systolic phase; IVSd, interventricular septal thickness at diastolic phase; LVPWs, left ventricular posterior wall thickness at systolic phase; LVPWd, left ventricular posterior wall thickness at diastolic phase; SV, stroke volume; EF, ejection fraction; FS, fractional shortening. *n* = 10 /group.
(DOC)
