# Supplementary figures and images for: Protective Effect of Sevoflurane Postconditioning against Cardiac Ischemia/Reperfusion Injury via Ameliorating Mitochondrial Impairment, Oxidative Stress and Rescuing Autophagic Clearance
Source: PLoS One. 2015 Aug 11;10(8):e0134666. doi: 10.1371/journal.pone.0134666 (PMC4532466; doi:10.1371/journal.pone.0134666)

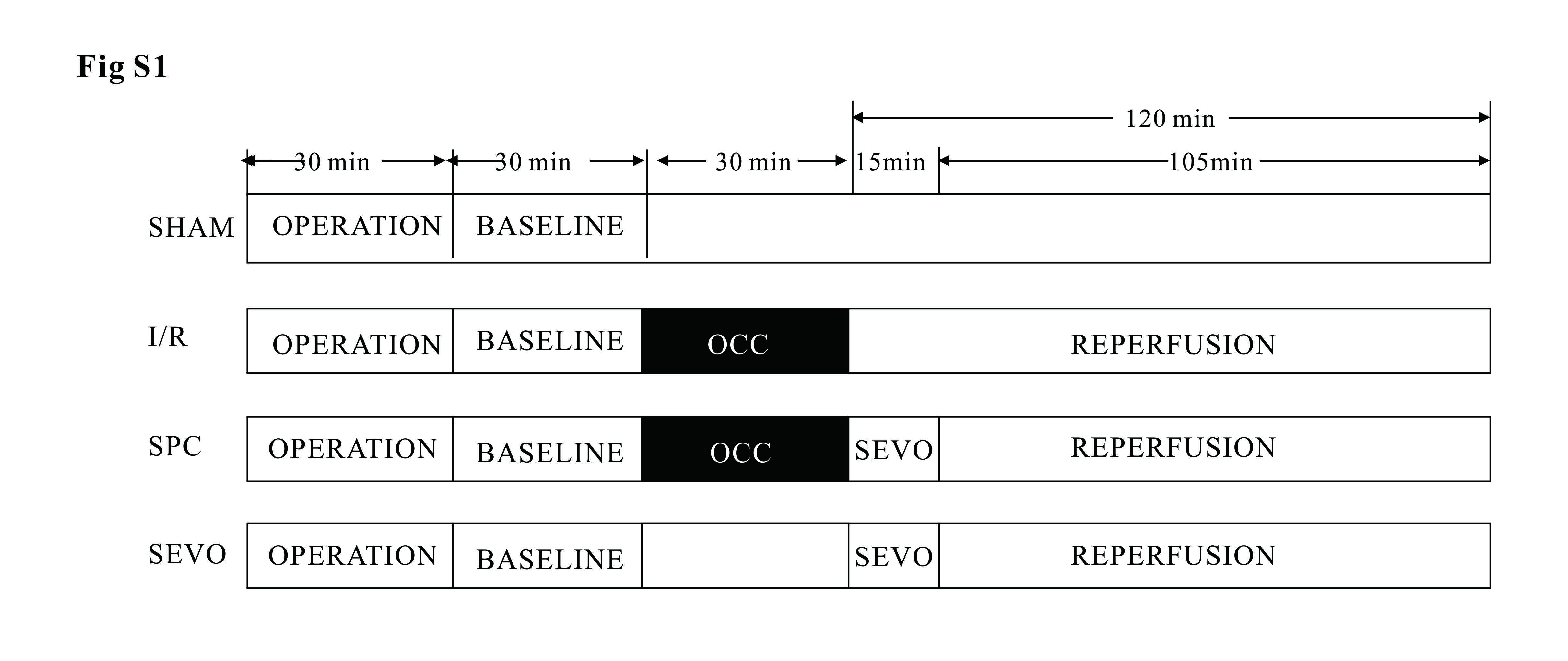

Supplement: S1 Fig — All the groups underwent the same surgical operation. (1) SHAM: rats were subjected to open chest surgery only; (2) I/R: rats were subjected to 30 min LAD occlusion, followed by 2 h of reperfusion; (3) SPC: rats were subjected to I/R and receiving 2.4% sevoflurane [1.0 minimum alveolar concentration (MAC) at 37°C] for 15 min at onset of reperfusion; (4) SEVO: rats received 1.0 MAC sevoflurane for 15 min without occlusion. (JPG) [file pone.0134666.s001.jpg]

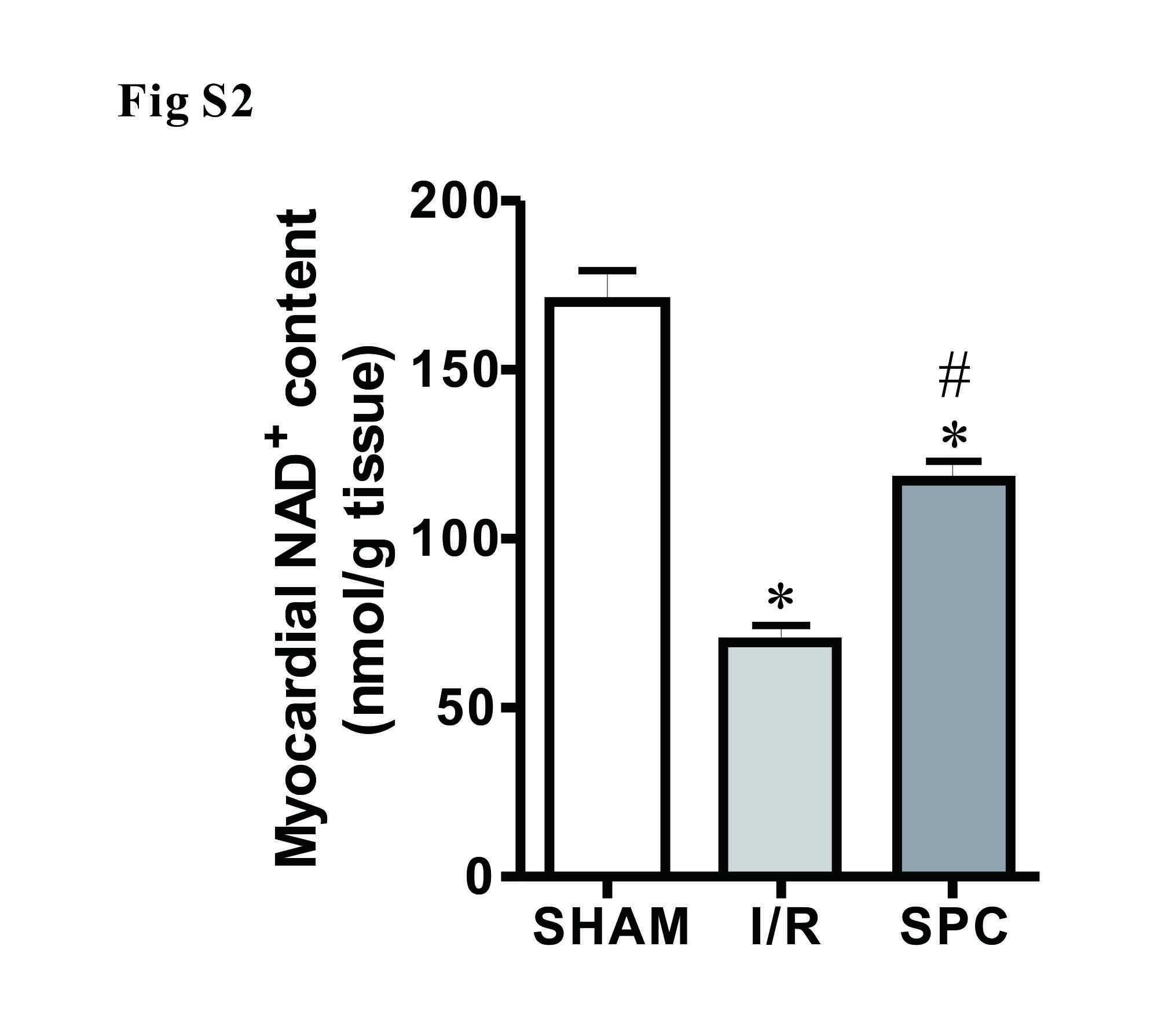

Supplement: S2 Fig — Compared with the SHAM group, the NAD+ content in the other groups decreased. And, the NAD+ content in the SPC group was significantly higher than I/R group, SPC prevents reducing of myocardial NAD+ content after I/R. n = 6 /group. NAD, nicotinamide adenine dinucleotide. (JPG) [file pone.0134666.s002.jpg]

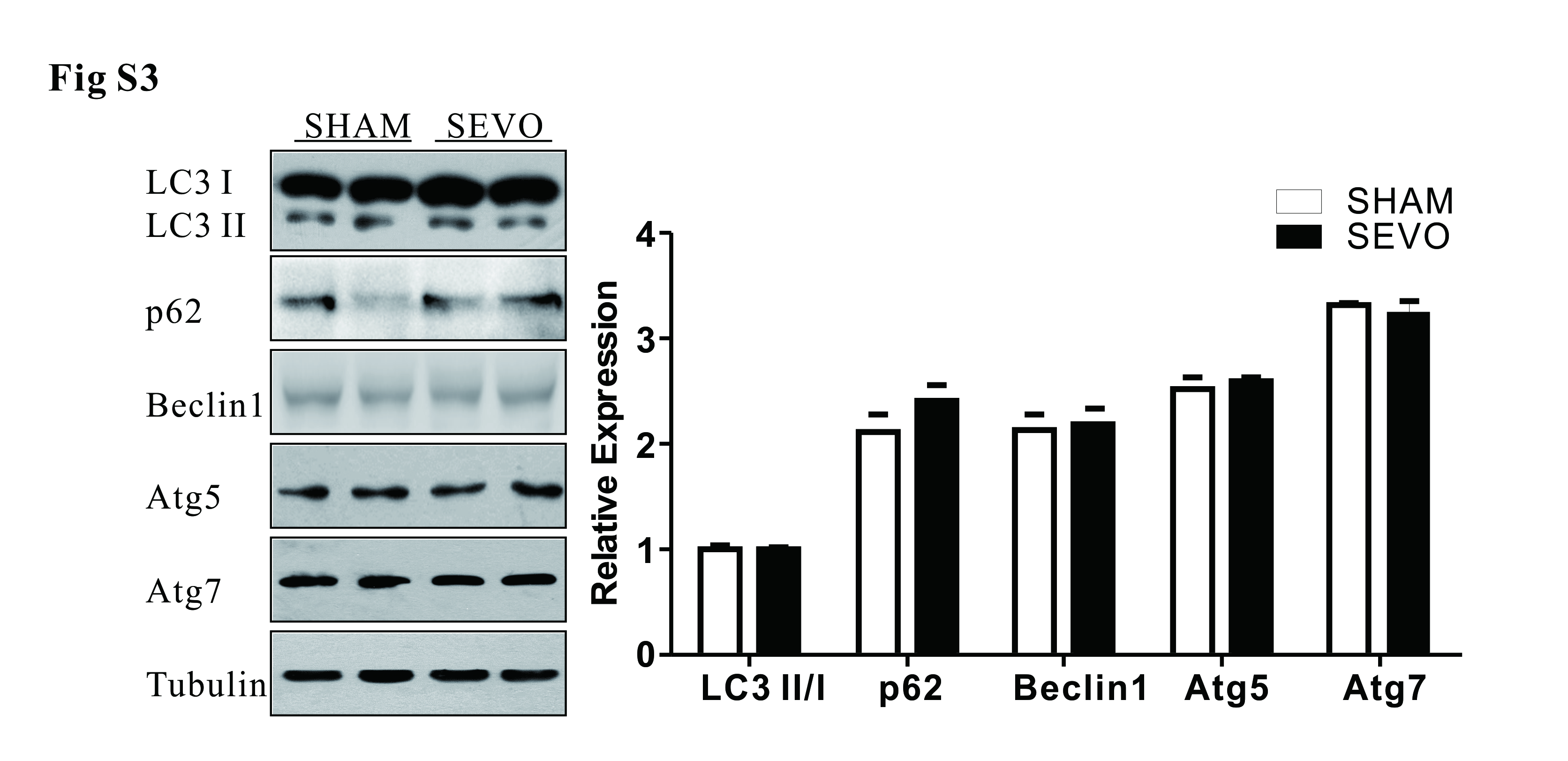

Supplement: S3 Fig — At the end of reperfusion, the autophagy associated proteins were compared between SHAM and SEVO group. Expressions of LC3 II/LC3 I ratio, Beclin1, Atg5 and Atg7 in SHAM and SEVO groups were measured by western blot. The autophagy associated proteins were no significant difference between SHAM group and SEVO group (P > 0.05). n = 4 /group. (JPG) [file pone.0134666.s003.jpg]

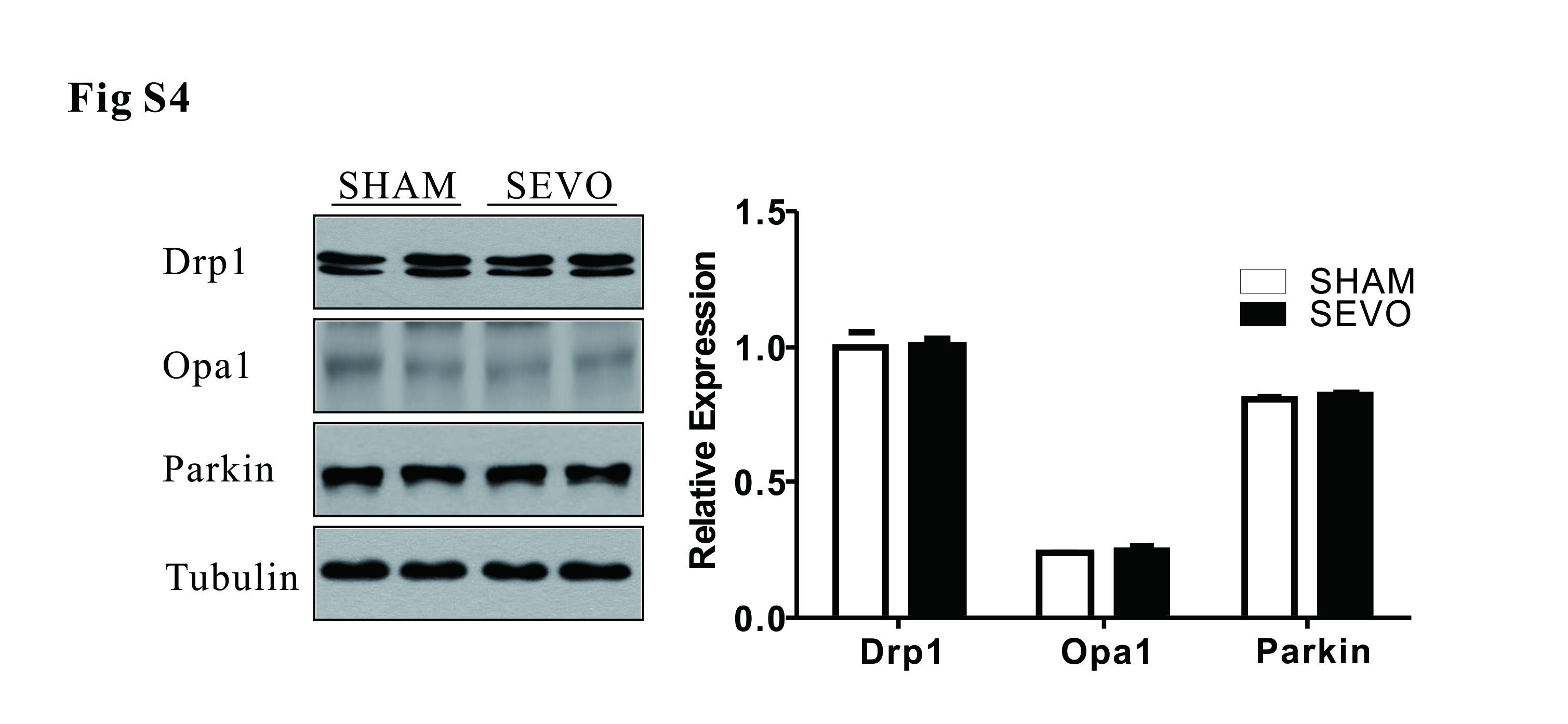

Supplement: S4 Fig — At the end of reperfusion, the mitophagy associated proteins were compared between SHAM and SEVO group. The expressions of Drp1, Opa1 and Parkin in SHAM and SEVO groups were measured by western blot. The mitophagy associated proteins were no significant difference between SHAM group and SEVO group (P > 0.05). n = 4 /group. (JPG) [file pone.0134666.s004.jpg]
