# Supplementary material for: Protective Effect of Sevoflurane Postconditioning against Cardiac Ischemia/Reperfusion Injury via Ameliorating Mitochondrial Impairment, Oxidative Stress and Rescuing Autophagic Clearance
Source: PLoS One. 2015 Aug 11;10(8):e0134666. doi: 10.1371/journal.pone.0134666 (PMC4532466; doi:10.1371/journal.pone.0134666)
Supplement: S1 File — (DOC) [file pone.0134666.s005.doc]

**Supporting Information**

**Antibodies and reagent**s

# Primary antibodies for Tubulin, Histone3(H3), superoxide dismutase 2 (SOD2), Heme Oxygenase 1 (HO-1), and lysosome associated membrane protein-2 (Lamp2) were from Bioworld (Minneapolis, MN), for peroxisome proliferator-activated receptor gamma coactivator (PGC)-1α, Vps34, phosphor-Akt (Ser473), Akt, phosphor-mTOR (Ser2448), microtubule-associated protein-1 light chain 3 (LC3), p62, Beclin1, autophagy-related gene 5 (Atg5), autophagy-related gene 7 (Atg7), Dynamin-related protein 1 (Drp1) and Parkin from Cell Signaling (Beverly, MA, USA), for nuclear respiratory factor (Nrf1), Vimentin and optic atrophy 1 gene protein (Opa1) from Santa Cruz (Santa Cruz, CA, USA). Analytical kits for adenosine triphosphate (ATP) content and malondialdehyde (MDA) activity were obtained from Jiancheng BioTech (Nanjing, China). Primary antibodies for reduced glutathione (GSH), oxidized glutathione (GSSG), 5-sulphosalisilic acid, 2-vinylpyridine, 2,3,5-triphenyltetrazolium chloride triazole (TTC) and the oxidant-sensitive fluorogenic probe dihydroethidium (DHE) were purchased from Sigma Aldrich (St. Louis, MO, USA). Hoechst 33342 reagent was from Invitrogen (Carlsbad, CA, USA) and the supersignal west pico chemiluminescent substrate was from Pierce (Rockford, IL, USA). The proteinase inhibitor cocktail was available from Roche Diagnostics GmbH (Mannheim, Germany).

**Measurement of mPTP opening**

In reperfusion period, myocardial NAD^+^ released from dysfunctional mitochondria while opening of the mPTP was increased. Therefore, myocardial NAD^+^ content is inversely proportional to mPTP opening, and myocardial NAD^+^ content is an indicator of mPTP opening[[^1^](#_ENREF_21)]. After 15 min of reperfusion, hearts were taken for measuring NAD^+^ content by the Klingenberg method as previously described[[^2^](#_ENREF_21)^-3]^. 30mg left ventricular tissue was powdered and mixed with perchloric acid. Then, the mixture was homogenized, neutralized and centrifuged. The centrifuged sediment was dissolved, and the dilutions of the supernatant samples were removed and NAD^+^ concentrations were determined fluorometrically using alcohol dehydrogenase (Sigma-Aldrich, St Louis, MO, USA) at a wavelength of 460 nm in a Multi-frequency Phase ISS K2 Spectrofluorimeter (ILC Technology nc, Sunnyvale, CA, USA). For measuring myocardial NAD^+^ content, six hearts were assessed in different experimental groups (*n* = 6 /group).

#

**References**

1 Feng J, Lucchinetti E, Ahuja P, Pasch T, Perriard JC, Zaugg M. Isoflurane postconditioning prevents opening of the mitochondrial permeability transition pore through inhibition of glycogen synthase kinase 3beta. Anesthesiology. 2005; 103: 987-995.

2 Xie H, Zhang J, Zhu J, Liu LX, Rebecchi M, Hu SM, *et al*. Sevoflurane post-conditioning protects isolated rat hearts against ischemia-reperfusion injury via activation of the ERK1/2 pathway. Acta Pharmacol Sin. 2014; 35: 1504-1513.
