# Supplementary material for: Protective Effect of Sevoflurane Postconditioning against Cardiac Ischemia/Reperfusion Injury via Ameliorating Mitochondrial Impairment, Oxidative Stress and Rescuing Autophagic Clearance
Source: PLoS One. 2015 Aug 11;10(8):e0134666. doi: 10.1371/journal.pone.0134666 (PMC4532466; doi:10.1371/journal.pone.0134666)
Supplement: S1 Table — Cycs, Cytochrome C; Cox4i1, Cytochrome c oxidase subunit 4 isoform 1; Ndufa2, NADH dehydrogenase (ubiquinone) 1 alpha subcomplex subunit 2; Ndufa4, NADH dehydrogenase (ubiquinone) 1 alpha subcomplex subunit 4; Ndufa8, NADH dehydrogenase (ubiquinone) 1 alpha subcomplex subunit 8; Cox7a1, Cytochrome c oxidase subunit VIIa polypeptide 1; Cox7a2, Cytochrome c oxidase subunit VIIa polypeptide 2; TFAM, transcription factor A mitochondrial. (DOC) [file pone.0134666.s006.doc]

**Supporting Table 1 Primers used in real-time PCR**

| Primers | Sequences |
| --- | --- |
| Cycs | forword:5-CCAAATCTCCACGGTCTGTTC-3  reverse: 5-ATCAGGGTATCCTCTCCCCAG-3 |
| Cox4i1 | forword:5-ATTGGCAAGAGAGCCATTTCTAC-3  reverse: 5-CACGCCGATCAGCGTAAGT-3 |
| Ndufa2 | forword:5-TTGCGTGAGATTCGCGTTCA-3  reverse: 5-ATTCGCGGATCAGAATGGGC-3 |
| Ndufa4 | forword: 5-TCCCAGCTTGATTCCTCTCTT-3  reverse: 5-GGGTTGTTCTTTCTGTCCCAG-3 |
| Ndufa8 | forword: 5-GGAGCTGCCAACTCTGGAAG-3  reverse: 5-CCAGCGGCACAGCATAAAC-3 |
| Cox7a1 | forword: 5-GCTCTGGTCCGGTCTTTTAGC-3  reverse: 5-GTACTGGGAGGTCATTGTCGG-3 |
| Cox7a2 | forword: 5-GCTGGCCCTTCGTCAGATT-3  reverse: 5-GGCATCCCATTATCCTCCTGAA-3 |
| TFAM | forword:5-ATTCCGAAGTGTTTTTCCAGCA-3  reverse: 5-TCTGAAAGTTTTGCATCTGGGT-3 |
| β-actin | forword:5-TAAAGACCTCTATGCCAACACAGT-3  reverse:5-CACGATGGAGGGGCCGGACTCATC-3 |
