# Supplementary material for: Protective Effect of Sevoflurane Postconditioning against Cardiac Ischemia/Reperfusion Injury via Ameliorating Mitochondrial Impairment, Oxidative Stress and Rescuing Autophagic Clearance
Source: PLoS One. 2015 Aug 11;10(8):e0134666. doi: 10.1371/journal.pone.0134666 (PMC4532466; doi:10.1371/journal.pone.0134666)
Supplement: S2 Table — * P <0.05 vs SHAM group; # P <0.05 vs I/R group. LVIDs, left ventricular internal diameter at systolic phase; LVIDd, left ventricular internal diameter at diastolic phase; IVSs, interventricular septal thickness at systolic phase; IVSd, interventricular septal thickness at diastolic phase; LVPWs, left ventricular posterior wall thickness at systolic phase; LVPWd, left ventricular posterior wall thickness at diastolic phase; SV, stroke volume; EF, ejection fraction; FS, fractional shortening. n = 10 /group. (DOC) [file pone.0134666.s007.doc]

**Supporting Table 2 Echocardiographic measurements 2 h after IR**

| Cardiac function | SHAM (*n* = 10) | I/R (*n* = 10) | I/R+SPC (*n* = 10) |
| --- | --- | --- | --- |
|
| LVIDs (mm) | 4.46±0.38 | 6.32±0.74^*^ | 5.25±0.32^*#^ |
| LVIDd (mm) | 6.68±0.56 | 7.69±0.69^*^ | 6.74±0.44^#^ |
| IVSs (mm) | 2.16±0.43 | 1.43±0.33^*^ | 1.69±0.35 |
| IVSd (mm) | 1.51±0.19 | 1.01±0.17^*^ | 1.33±0.16^*#^ |
| LVVs (ml) | 100.31±25.63 | 209.43±16.57^*^ | 137.16±19.39^*#^ |
| LVVd (ml) | 259.29±32.91 | 305.18±17.89^*^ | 251.42±25.89^#^ |
| LVPWs (mm) | 2.04±0.27 | 1.39±0.24^*^ | 1.62±0.54^*^ |
| LVPWd (mm) | 1.61±0.22 | 1.30±0.45 | 1.40±0.27 |
| SV (ml) | 158.98±11.41 | 95.75±8.01^*^ | 114.26±12.76^*#^ |
| EF (%) | 64.89±1.91 | 31.47±1.71^*^ | 44.72±4.19^*#^ |
| FS (%) | 34.98±1.45 | 16.83±2.60^*^ | 23.43±3.20^*#^ |

** P* <0.05 vs SHAM group; # *P* <0.05 vs I/R group. LVIDs, left ventricular internal diameter at systolic phase; LVIDd, left ventricular internal diameter at diastolic phase; IVSs, interventricular septal thickness at systolic phase; IVSd, interventricular septal thickness at diastolic phase; LVPWs, left ventricular posterior wall thickness at systolic phase; LVPWd, left ventricular posterior wall thickness at diastolic phase; SV, stroke volume; EF, ejection fraction; FS, fractional shortening. *n* = 10 /group.
